# Serum vitamin D as a novel biomarker for asymptomatic uterine fibroids: re-evaluating the risk threshold for incident fibroid development

**DOI:** 10.3389/fgwh.2025.1665791

**Published:** 2025-12-01

**Authors:** Nenghuan Tang, Haoran Li, Can Luo, Li Hu, Chunling Fang, Jie Yu, Fan Xu

**Affiliations:** 1 Department of Gynecology, Shanghai First Maternity and Infant Hospital, School of Medicine, Tongji University, Shanghai, China; 2 Department of Obstetrics and Gynecology, Affiliated Hospital of North Sichuan Medical College, Nanchong, Sichuan, China; 3 North Sichuan Medical College, Nanchong, Sichuan, China; 4 Department of Obstetrics and Gynecology, the Affiliated Nanchong Central Hospital of North Sichuan Medical College, Nanchong, Sichuan, China

**Keywords:** vitamin D, threshold, asymptomatic uterine fibroids, Han Chinese women, biomarker

## Abstract

**Objective:**

Lower serum vitamin D levels are inversely correlated with the risk of uterine fibroids (UFs). The existing vitamin D deficiency cutoffs are defined based on the risk of bone loss instead of asymptomatic fibroids. This study aimed to clarify the relationship between vitamin D deficiency and first-time diagnosed asymptomatic UFs, and identify the optimal threshold for assessing the risk of newly-developed fibroids.

**Methods:**

In this case-control study, 171 asymptomatic UFs patients and 125 normal controls aged 34–45 were enrolled. A total of 64 pairs matched by age, BMI, gravity and parity were created. Logistic regression models were used to examine the correlation between vitamin D and the risk of asymptomatic UFs, and the receiver operating characteristic curve was employed to evaluate the diagnostic performance of vitamin D deficiency thresholds for the risk of asymptomatic fibroids.

**Results:**

Both in the unmatched and matched groups, serum vitamin D levels of asymptomatic patients were lower than those of normal control individuals [(12.79 ± 5.55 vs. 16.19 ± 4.64, *p* < 0.001) and (11.95 ± 5.27 vs. 17.29 ± 4.99, *p* < 0.001)], and multivariate logistic regression revealed that vitamin D deficiency was related to an increased risk of asymptomatic UFs [(OR = 0.81; 95% Cl = 0.76–0.87) and (OR = 0.79; 95% Cl = 0.72–0.87)]. Compared to the widely-used vitamin D deficiency thresholds (12.00 ng/mL, 20.00 ng/mL and 30.00 ng/mL), the new cutoff of 14.34 ng/mL achieved the highest sensitivity and specificity to asymptomatic UFs (sensitivity 70.3%, specificity 76.6%, accuracy: 73.4%).

**Conclusions:**

The newly-developed vitamin D deficiency threshold pf 14.34 ng/mL generates a balanced screening efficacy in women at a higher risk of UFs, and provides a possible approach for the secondary prevention strategy of UFs.

## 1 Introduction

Uterine fibroids (UFs) are the most common benign tumors of the uterus, and at least 50% of fibroids are asymptomatic (1). Despite the high prevalence in premenopausal women, a large proportion of women with fibroids are unaware of the asymptomatic tumors and do not seek for further medical care (2). Because of failing to conduct a timely detection and early intervention, fibroids may grow and then become symptomatic in the future (3–5), even causing a damage to fertility and affecting obstetric outcomes, such as preterm delivery and lower birthweight infants (6). However, compared to available management and treatment strategy for symptomatic patients, the asymptomatic individuals have not received due attention (7). To avoid the continuous development of asymptomatic fibroids to a size at which symptoms appear or treatment becomes more challenging, it is of great significance to develop a secondary prevention strategy for evaluating the risk of newly-developed UFs.

Numerous studies have demonstrated that vitamin D plays an important role in the development of UFs, and setting a screening strategy and guideline of vitamin D deficiency for identifying the population with a high risk of fibroids may be of potential importance to the secondary prevention of UFs (8, 9). However, asymptomatic patients are not particularly emphasized and analyzed among those studies, leading to a lack of evidence for the recommendation of vitamin D management in asymptomatic patients. Additionally, there is no consensus on the optimal levels of serum 25(OH)D, particularly for the risk of newly-developed UFs (10). The state of vitamin D deficiency is usually defined by the serum 25(OH)D concentrations below 12.00 ng/mL, 20.00 ng/mL or 30.00 ng/mL, which are relevant to the risk of bone loss (11–14). In contrast, our previous study firstly reported that the serum 25(OH)D concentration below 14.34 ng/mL was featured with a satisfied accuracy, balanced sensitivity and specificity in assessing the risk of newly-developed fibroid growth (15). Whereas, there is no evidence to support the threshold value that is capable for assessing the risk of newly-developed fibroids. Thus, it is necessary to clarify the relationship between vitamin D deficiency and the risk of first-time diagnosed asymptomatic UFs, and re-visit a key threshold adopted to evaluate the risk of newly-developed fibroids.

Therefore, in this paper, a case-control study was designed, aiming to: (1) provide updated data to validate the relationship between vitamin D and newly-developed asymptomatic UFs; and (2) identify a better vitamin D threshold for assessing the risk of asymptomatic fibroids in Han Chinese women.

## 2 Materials and methods

### 2.1 Study participants

In this case control study, we enrolled 171 premenopausal Chinese Han women diagnosed unexpectedly with asymptomatic UFs through routine gynecological ultrasonography at the Health Management Center and Gynecology Outpatient Department of Nanchong Central Hospital from January 2023 to March 2024. At the same time, 125 healthy control subjects were recruited by the Health Management Center in Nanchong Central Hospital. All patients signed the informed written consent, and the study was approved by the Ethics Committee of Nanchong Central Hospital, the Second Clinical Medical College, North Sichuan Medical College [Number: (2022076)].

Inclusion criteria: (1) for patients diagnosed with asymptomatic UFs: (a) premenopausal Chinese Han women who were newly diagnosed with UFs by both transabdominal and transvaginal ultrasound examinations, and (b): UFs patients without symptoms including bulk symptoms, chief complaint of abnormal menstrual bleeding, pelvic pain or anemia; and (2) for healthy control individuals: (a): premenopausal Chinese Han women without UFs or other gynecological diseases, and (b): those diagnosed without diseases related to vitamin D.

Exclusion criteria: (1): for patients with symptoms of UFs: (a) menopausal women, (b) patients who had been previously diagnosed with or treated for UFs, (c) patients who had taken vitamin D supplement within the past year; and (2) for healthy control individuals: (a) menopausal women, (b) women who had been previously diagnosed with UFs or other diseases associated with vitamin D, and (c): those with concurrent tumors or other gynecological diseases.

### 2.2 Detection methods

Blood samples of all participants were collected into the red-top vacuum tubes containing silica clot activator (Becton, Dickinson and Company). Serum samples were stored at −20 °C and then assayed for less than 7 days. The serum 25(OH)D level was measured by Elecsys Vitamin D total (Elecsys and cobas e immunoassay analyzers). Serum LDH concentration was detected by immunoradiometric assay. Clinical and anthropometric variables including the body mass index (BMI), gravidity, parity, and age were collected in all individuals.

### 2.3 Data analysis

Between the UFs and normal control groups, 1:1 propensity score matching was conducted on age (±1 year), parity (≤1 vs. >1), gravidity (≤3 vs. >3) and BMI (≤24 vs. >24). To minimize the bias, cases were randomly ordered before matching, which was performed without replacement using the optimal matching algorithm in SPSS (Version 26.0), and 64 matched pairs were obtained. According to the World Health Organization classification for the Chinese population, BMI was classified into >24 or ≤24 kg/m^2^ (16). Based on the threshold presented in our previous study, serum vitamin D concentration was categorized into ≤14.34 ng/mL or >14.34 ng/mL (15). According to previous research, parity was classified into >1 or ≤1, gravidity into >3 or ≤3, age into >40 or ≤40, and LDH into >170 or ≤170 U/L (17, 18).

The data were analyzed by the statistical software SPSS26.0. Vitamin D level, age, BMI, gravidity, parity, and LDH between the UFs and normal control groups were compared using the Chi-square and t test. For the unmatched groups, unconditional logistic regression was performed to estimate odds ratios (OR) and 95% confidence intervals (CI) for vitamin D and UFs, adjusting for gravidity, parity, age, BMI and LDH. Moreover, conditional logistic regression was used in the matched groups. The diagnostic value of the vitamin D levels 12.00 ng/mL, 14.34 ng/mL, 20 ng/mL and 30 ng/mL was determined on the base of the receiver operating characteristics (ROC) curve. The two-sided *p*-value < 0.05 was considered statistically significant.

## 3 Results

### 3.1 Baseline characteristics in the matched and unmatched groups

A total of 171 patients with asymptomatic UFs and 125 healthy control women were involved for this analysis. Table 1 shows the baseline characteristics of the study population. In the unmatched groups, the mean vitamin D level (ng/mL) of UFs patients was significantly lower than that of healthy control individuals (12.79 ± 5.55 vs. 16.19 ± 4.64, *p* < 0.001). Apart from that, the UFs group had an elevated mean age (44.19 ± 6.41 vs. 34.82 ± 9.07, *p* < 0.001), and higher gravidity and parity (*p* < 0.001). In the matched groups, a total of 128 subjects were included in the final analysis (64 UFs patients matched at 1:1–64 healthy control individuals). No significant differences were found in baseline characteristics between the two groups (*p* > 0.05). In the matched population, the mean vitamin D level (ng/mL) of UFs patients was obviously lower than that of healthy control individuals (11.95 ± 5.27 vs. 17.29 ± 4.99, *p* < 0.001).

**Table 1:** Clinical characteristics of patients in unmatched and matched groups.

Characteristic	Unmatched groups			Matched groups		
	Normal group	UFs group	*P* value	Normal group	UFs group	*P* value
	(*n* = 125)	(*n* = 171)		(*n* = 64)	(*n* = 64)	
Vitamin D level, ng/mL	16.19 ± 4.64	12.79 ± 5.55	<0.001	17.29 ± 4.99	11.95 ± 5.27	<0.001
Age, y	34.82 ± 9.07	44.19 ± 6.41	<0.001	40.81 ± 7.98	40.53 ± 8.32	0.85
Age, y, *n* (%)						
≤40	94 (75.2%)	33 (19.3%)	<0.001	33 (51.6%)	26 (40.6%)	0.22
>40	31 (24.8%)	138 (80.7%)		31 (48.4%)	38 (59.4%)	
BMI, kg/m^2^	22.88 ± 3.32	24.07 ± 3.19	0.002	23.09 ± 3.69	23.15 ± 3.23	0.92
BMI, kg/m^2^, *n* (%)						
≤24	86 (68.8%)	88 (51.5%)	0.003	42 (65.6%)	43 (67.2%)	0.85
>24	39 (31.2%)	83 (48.5%)		22 (34.4%)	21 (32.8%)	
Gravidity, *n* (%)						
≤3	103 (82.4%)	109 (63.7%)	<0.001	50 (78.1%)	44 (68.8%)	0.23
>3	22 (17.6%)	62 (36.3%)		14 (21.9%)	20 (31.3%)	
Parity, *n* (%)						
≤1	84 (67.2%)	74 (43.3%)	<0.001	38 (59.4%)	31 (48.4%)	0.22
>1	41 (32.8%)	97 (56.7%)		26 (40.6%)	33 (51.6%)	
LDH, U/L	164.20 ± 26.60	169.32 ± 30.82	0.14	163.63 ± 28.40	165.73 ± 30.60	0.69
LDH, U/L, *n* (%)						
≤170	74 (59.2%)	95 (55.6%)	0.53	40 (62.5%)	37 (57.8%)	0.59
>170	51 (40.8%)	76 (44.4%)		24 (37.5%)	27 (42.2%)	

BMI, body mass index; LDH, lactate dehydrogenase; UFs, uterine fibroid.

Data are presented as mean SD, or number (%). *T* test for continuous variables and chi- square test for categorical variables.

Case and control subjects were matched by age, BMI, gravity and parity.

### 3.2 Multivariable logistic regression for the presence of UFs

In the unmatched groups, the multivariate logistic regression indicated that lower serum vitamin D levels were associated with a higher risk of asymptomatic UFs [odds ratio (OR) 0.81, 95% CI 0.76–0.87; *P* < .001], and higher age and gravidity were related to an elevated risk of asymptomatic UFs [odds ratio (OR): 1.18, 95% CI: 1.13–1.24, *P* < 0.001 and odds ratio (OR): 1.31, 95% CI: 1.05–1.63, *P* < 0.05 respectively]. In the matched groups, a similar relationship between vitamin D level and asymptomatic UFs [odds ratio (OR) 0.79, 95% CI 0.72–0.87; *P* < .001] was observed (Table 2).

**Table 2:** Multivariable logistic regression for the presence of UFs in unmatched and matched groups.

Variable	Unmatched groups			Matched groups		
	(*n* = 296)			(*n* = 128)		
	OR	95% Cl	*P* value	OR	95% Cl	*P* value
Vitamin D level	0.81	0.76–0.87	<0.001	0.79	0.72–0.87	<0.001
Age	1.18	1.13–1.24	<0.001	1.01	0.95–1.08	0.70
Gravidity	1.31	1.05–1.63	0.02	1.12	0.85–1.48	0.41
BMI	1.02	0.92–1.12	0.69	0.96	0.84–1.09	0.54
Parity	0.88	0.56–1.39	0.58	1.42	0.77–2.61	0.27
LDH	0.99	0.98–1.01	0.79	1.00	0.99–1.02	0.67

BMI, body mass index; LDH, lactate dehydrogenase; UFs, uterine fibroid.

Data are presented as mean SD, or number (%). *T* test for continuous variables and chi-square test for categorical variables.

Case and control subjects were matched by age, BMI, gravity and parity.

### 3.3 Diagnostic value of different vitamin D thresholds for asymptomatic UFs

Compared with other vitamin D deficiency thresholds associated with the risk of skeletal disease, the threshold of 14.34 ng/mL generated the most balanced predictive performance (AUC: 0.8, 95% Cl: 0.7–0.9, sensitivity: 70.3%, specificity: 76.6%, PPV: 75.0%, NPV: 72.1%, accuracy: 73.4%) in the matched groups (*n* = 128). The predictive statistics for the level of 12.00 ng/mL were: AUC: 0.8, 95% Cl: 0.7–0.9, sensitivity: 54.7%, specificity: 87.5%, PPV: 81.4%, NPV: 65.9%, accuracy: 71.1%; those for 20.00 ng/mL were: AUC: 0.8, 95% Cl: 0.7–0.9, sensitivity: 95.3%, specificity: 25.0%, PPV: 55.9%, NPV: 84.2%, and accuracy: 60.2%; while those for 30.00 ng/mL, were: AUC: 0.8, 95% Cl: 0.7–0.9, sensitivity: 98.4%, specificity: 1.6%, PPV: 50.0%, NPV: 50.0%, and accuracy: 50.8% (Table 3).

**Table 3:** Diagnostic value of vitamin D for asymptomatic UFs in matched groups (*n* = 128).

Vitamin D threshold								
Statistic	12.00 ng/mL		14.34 ng/mL		20.00 ng/mL		30.00 ng/mL	
	Value	95% CI	Value	95% CI	Value	95% CI	Value	95% CI
AUC	0.8	0.7–0.9	0.8	0.7–0.9	0.8	0.7–0.9	0.8	0.7–0.9
Sensitivity, %	54.7	41.8–67.0	70.3	57.4–80.8	95.3	86.0–98.8	98.4	90.5–99.9
Specificity, %)	87.5	76.3–94.1	76.6	64.0–85.9	25.0	15.4–37.7	1.6	0.1–9.5
PPV, %	81.4	66.1–91.1	75.0	61.9–84.9	55.9	46.1–65.4	50.0	41.0–58.9
NPV, %	65.9	54.7–75.6	72.1	59.7–81.9	84.2	59.5–95.8	50.0	2.7–97.3
LR+	4.4	2.2–8.7	3.0	1.9–4.8	1.3	1.1–1.5	1.0	0.9–1.1
LR−	0.5	0.4–0.7	0.4	0.3–0.6	0.2	0.1–0.6	1.0	0.02–48.1
Accuracy, %	71.1	–	73.4	–	60.2	–	50.8	–

AUC, area under the receiver operating characteristic curve; CI, confidence interval; LR+, positive likelihood ratio [sensitivity/(1- specificity)]; LR−, negative likelihood ratio [(1—sensitivity)/specificity]; NPV, negative predictive value; PPV, positive predictive value.

Case and control subjects were matched by age, BMI, gravity and parity.

## 4 Discussion

To the best of our knowledge, an optimally validated threshold in relation with the risk of asymptomatic UFs has not been addressed in previous studies. In this case-control study of premenopausal Han Chinese participants, it was observed that asymptomatic UFs patients were inclined to having vitamin D deficiency compared to the healthy control individuals. Then, the predictive performance of all current vitamin D deficiency thresholds (12.00 ng/mL, 14.34 ng/mL, 20.00 ng/mL, 30.00 ng/mL) was re-evaluated, and it was indicated that the cutoff point of 14.34 ng/mL generated the most balanced predictive value for asymptomatic UFs in the updated data. Admittedly, this is the first study that reports a validated threshold particularly for assessing the risk of new fibroid growth.

UFs are one of the most common benign tumors of the uterus, and asymptomatic patients account for a large proportion of the population. Compared to symptomatic subjects, asymptomatic patients are more likely to be ignored. Therefore, it is important to develop a prevention strategy for disease management and risk evaluation of newly formed fibroid. Vitamin D deficiency has been demonstrated to be associated with the elevated risk of UFs (19–22). In our previous study, it was also assessed that lower Vitamin D level increased the risk of newly-developed fibroid (15), and the present study further validated the relationship between the vitamin D level and the risk of newly developed fibroids. In other words, the vitamin D level can be used as a key factor for the development of a secondary prevention strategy.

The thresholds of 25(OH)D serum concentrations below 12.00 ng/mL, 20.00 ng/mL or 30.00 ng/mL are generated from the population with bone diseases (23, 24), and our previous study firstly provided a cut-off point below 14.34 ng/mL for newly-developed UFs (15). However, the evidence to support which threshold value is optimal for UFs is lacking. Thus, these thresholds need to be re-evaluated. In our present study, the threshold of 14.34 ng/mL performed the highest accuracy compared with others (12.00 ng/mL, 20.00 ng/mL or 30.00 ng/mL). Other than that, the cut-off value of 12.00 ng/mL had higher specificity but very low sensitivity than those of 14.34 ng/mL. The thresholds of 20.00 ng/mL and 30.00 ng/mL featured higher sensitivity but poor specificity than those of 14.34 ng/mL. Therefore, the cut-off value of 14.34 ng/mL generated the most balanced predictive value for newly-developed UFs. This threshold is conductive to the establishment of screening strategies for women at a high risk of UFs, and further secondary prevention to achieve early detection and intervention of newly developed fibroids.

The biological mechanism of vitamin D in UFs development has been proved in several studies (25, 26). It is revealed that vitamin D reduces the growth of immortalized human uterine fibroid cells (HuLM), which is related to the down-regulation of cell proliferation nuclear antigen (PCNA), beta-cell lymphoma 2 (Bcl-2), Bcl-w, cyclin-dependent kinase 1 (CDK1) and catechol-O-methyltransferase (COMT) (27). In addition, vitamin D may affect the expression of progesterone (PR) and estrogens (ER) to regulate the development of UFs (28). The experimental data also support that vitamin D affects the wingless-type(Wnt)/β-catenin pathway of UFs (29–31).

Our study has the following limitations. Firstly, for further clinical implementation, this threshold should be validated in the real-world data with the larger population. Secondly, as our study samples were restricted to premenopausal Chinese Han women, with relatively few participants aged 20–30 years, our results may not be applicable to all groups. Considering that, populations of different ages and races/ethnicities are required to re-evaluate their specific vitamin D deficiency threshold levels for UFs. Thirdly, sun exposure and dietary factors may have an influence on serum vitamin D level. However, the selection of our population was concentrated in one particular region (Nanchong), which means slight difference in the exposure of sunshine and the dietary pattern among subjects. Future studies should further examine those unmeasured factors. Fourthly, the measurement bias in detecting the vitamin D level could not be eliminated completely. Nonetheless, our present study provides a validated threshold to support the development of screening strategies for women at a high risk of UFs.

In conclusion, for evaluating the risk of newly-developed fibroids, the threshold of 14.34 ng/mL provides satisfied accuracy and achieves a balanced predictive value compared to other widely-used vitamin D deficiency cut-off points in premenopausal Han Chinese women. The value supports the potential criteria for screening strategies in the UFs high risk population, and is conductive to achieving early detection and intervention for newly-developed fibroids.

## Data Availability

The raw data supporting the conclusions of this article will be made available by the authors, without undue reservation.
